# 6-Chloro-1-({[(2*E*)-2-methyl-3-phenyl­prop-2-en-1-yl]­oxy}meth­yl)-1,2,3,4-tetra­hydro­quinazoline-2,4-dione

**DOI:** 10.1107/S1600536812020405

**Published:** 2012-05-19

**Authors:** Nasser R. El-Brollosy, Mohamed I. Attia, Ali A. El-Emam, Seik Weng Ng, Edward R. T. Tiekink

**Affiliations:** a Department of Pharmaceutical Chemistry, College of Pharmacy, King Saud University, Riyadh 11451, Saudi Arabia; b Department of Chemistry, Faculty of Science, Tanta University, Tanta 31527, Egypt; c Department of Chemistry, University of Malaya, 50603 Kuala Lumpur, Malaysia; d Chemistry Department, Faculty of Science, King Abdulaziz University, PO Box 80203 Jeddah, Saudi Arabia

## Abstract

In the title compound, C_19_H_17_ClN_2_O_3_, the conformation about the ethyl­ene bond [1.333 (2) Å] is *E*. The ten atoms comprising the quinazoline ring are essentially planar (r.m.s. deviation = 0.032 Å) and their mean plane forms a dihedral angle of 13.89 (7)° with the terminal phenyl ring; the mol­ecule has an open conformation as these substituents are directed away from each other. In the crystal, centrosymmetrically related mol­ecules are connected *via* N—H⋯O hydrogen bonds between the amide groups, leading to eight-membered {⋯HNCO}_2_ synthons. These are consolidated into a three-dimensional architecture by C—H⋯O, C—H⋯π and π–π inter­actions [ring centroid(N_2_C_4_)⋯centroid(C_6_) distance = 3.5820 (11) Å].

## Related literature

For background to non-nucleoside reverse transcriptase inhib­itors, see: Hopkins *et al.* (1996 ▶, 1999 ▶); El-Brollosy *et al.* (2008 ▶, 2009 ▶). For a related structure, see: El-Brollosy *et al.* (2012 ▶). For the synthesis, see: El-Brollosy (2007 ▶).
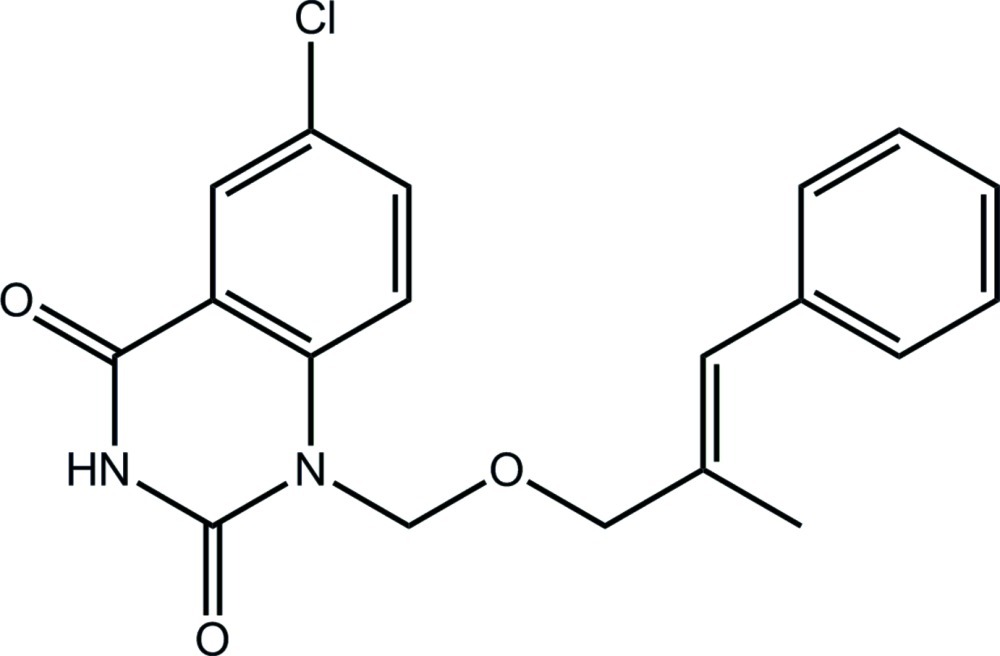


## Experimental

### 

#### Crystal data

C_19_H_17_ClN_2_O_3_
*M* *_r_* = 356.80
Triclinic, 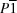

*a* = 7.6179 (3) Å
*b* = 9.8168 (4) Å
*c* = 11.7009 (6) Å
α = 73.937 (4)°
β = 83.651 (3)°
γ = 80.942 (3)°
*V* = 828.31 (6) Å^3^
*Z* = 2
Mo *K*α radiation
μ = 0.25 mm^−1^
*T* = 100 K
0.35 × 0.30 × 0.15 mm

#### Data collection

Agilent SuperNova Dual diffractometer with an Atlas detector
Absorption correction: multi-scan (*CrysAlis PRO*; Agilent, 2011 ▶) *T*_min_ = 0.904, *T*_max_ = 1.000
13263 measured reflections
3817 independent reflections
3107 reflections with *I* > 2σ(*I*)
*R*_int_ = 0.040

#### Refinement

*R*[*F* ^2^ > 2σ(*F* ^2^)] = 0.041
*wR*(*F* ^2^) = 0.114
*S* = 1.04
3817 reflections
231 parameters
H atoms treated by a mixture of independent and constrained refinement
Δρ_max_ = 0.35 e Å^−3^
Δρ_min_ = −0.27 e Å^−3^

### 

Data collection: *CrysAlis PRO* (Agilent, 2011 ▶); cell refinement: *CrysAlis PRO*; data reduction: *CrysAlis PRO*; program(s) used to solve structure: *SHELXS97* (Sheldrick, 2008 ▶); program(s) used to refine structure: *SHELXL97* (Sheldrick, 2008 ▶); molecular graphics: *ORTEP-3* (Farrugia, 1997 ▶) and *DIAMOND* (Brandenburg, 2006 ▶); software used to prepare material for publication: *publCIF* (Westrip, 2010 ▶).

## Supplementary Material

Crystal structure: contains datablock(s) global, I. DOI: 10.1107/S1600536812020405/hg5225sup1.cif

Structure factors: contains datablock(s) I. DOI: 10.1107/S1600536812020405/hg5225Isup2.hkl

Supplementary material file. DOI: 10.1107/S1600536812020405/hg5225Isup3.cml

Additional supplementary materials:  crystallographic information; 3D view; checkCIF report

## Figures and Tables

**Table 1:** Hydrogen-bond geometry (Å, °) *Cg*1 is the centroid of the C14–C19 benzene ring.

*D*—H⋯*A*	*D*—H	H⋯*A*	*D*⋯*A*	*D*—H⋯*A*
N1—H1*n*⋯O2^i^	0.85 (2)	2.05 (2)	2.8932 (18)	168.9 (19)
C4—H4⋯O1^ii^	0.95	2.57	3.382 (2)	144
C5—H5⋯O3^iii^	0.95	2.57	3.427 (2)	150
C9—H9*B*⋯O1^ii^	0.99	2.38	3.232 (2)	144
C10—H10*A*⋯*Cg*1^iv^	0.99	2.69	3.612 (2)	154

Symmetry codes: (i) 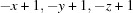; (ii) 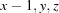; (iii) 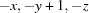; (iv) 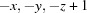.

**Fig. 1.**
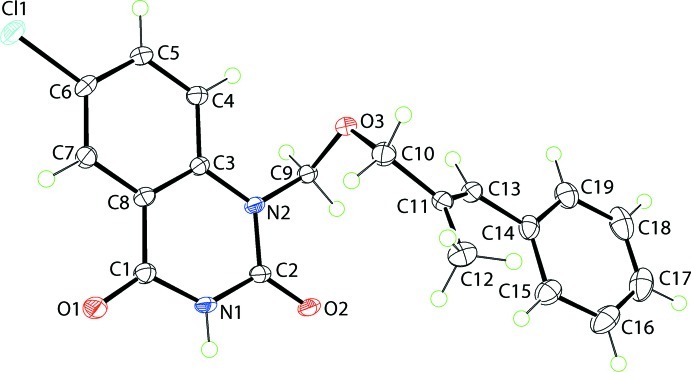
The molecular structure of (I) showing the atom-labelling scheme and displacement ellipsoids at the 50% probability level.

**Fig. 2.**
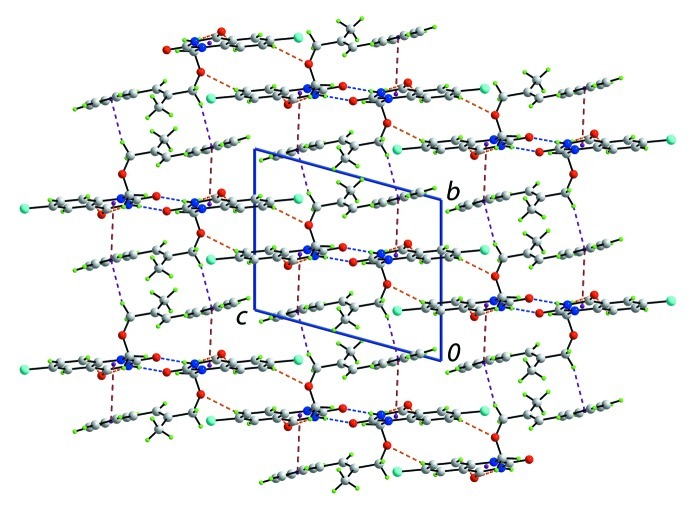
A view in projection down the a axis of the unit-cell contents for (I). The N—H···O, C—H···O, C—H···π and π—π interactions are shown as blue, orange, purple and brown dashed lines, respectively.

## supplementary crystallographic information

### Comment

Non-nucleoside reverse transcriptase inhibitors (NNRTI's) are very promising therapies in the treatment of human immunodeficiency virus (HIV) (Hopkins *et al.*, 1996; Hopkins *et al.*, 1999). In continuation to our interest in the chemistry of NNRTI's (El-Brollosy *et al.*, 2008; El-Brollosy *et al.*, 2009), we synthesized the title compound, 6-chloro-1-[((*E*)-2-methyl-3-phenylallyloxy)methyl]quinazoline-2,4(1*H*,3*H*)-dione (I), as a potential NNRTI (El-Brollosy, 2007). Herein, we describe the results of its crystal structure determination and relate this to the structure of the recently determined methyl analogue (El-Brollosy *et al.*, 2012).

In (I), Fig. 1, the conformation about the ethylene bond [C11═C13 = 1.333 (2) Å] is *E*. The 10 atoms comprising the quinazoline ring are planar with a r.m.s. = 0.032 Å; the maximum deviations from the least-squares plane = 0.051 (2) Å for the C1 atom and -0.046 (2) Å for the C2 atom. The dihedral angle between the fused ring system and the terminal phenyl ring of 13.89 (7)° is consistent with a twisted molecule; these substituents are directed away from each other so that the molecule has an open conformation. The torsion angle between the ethylene and phenyl rings, *i.e*. C11—C13—C14—C15, of 25.9 (3)° indicates a significant twist in this region of the molecule. However twisted the molecule of (I) is, it is less twisted than the methyl analogue where the dihedral angle between the quinazoline and phenyl rings was found to be 82.87 (7)° (El-Brollosy *et al.*, 2012).

Centrosymmetrically related molecules are connected *via* N—H···O hydrogen bonds between the amide groups (involving the carbonyl-O closest to the tertiary-N atom) and lead to eight-membered {···HNCO}_2_ synthons, Table 1. These are consolidated into a three-dimensional architecture by C—H···O and C—H···π interactions, Table 1, and π—π contacts [ring centroid(N1,N2,C1–C3,C8)···centroid(C14–C19)^i^ = 3.5820 (11) Å and tilt angle = 13.17 (9)°, for symmetry operation *i*: -*x*, 1 - *y*, 1 - *z*). Globally, the crystal structure comprises alternating layers of quinazoline rings and 2-methyl-3-phenylallyloxy)methyl residues that stack along the *b* axis, Fig. 2.

### Experimental

6-Chloroquinazoline-2,4(1*H*,3*H*)-dione (0.197 g, 1 mmol) was stirred in dry acetonitrile (15 ml) under nitrogen and *N*,*O*-bis(trimethylsilyl)acetamide (0.87 ml, 3.5 mmol) added. After a clear solution was obtained (10 min), the mixture was cooled to 223 K, and trimethylsilyl trifluoromethanesulfonate (0.18 ml, 1 mmol) was added followed by the drop wise addition of bis[(*E*)-2-methyl-3-phenylallyloxy]methane (0.616 g, 2 mmol). The reaction mixture was stirred at room temperature for 5 h. The reaction was quenched by the addition of saturated *aq*. NaHCO_3_ solution (5 ml). The mixture was evaporated under reduced pressure and the residue was extracted with ether (3 × 50 ml). The combined ether fractions were collected, dried (MgSO_4_) and evaporated under reduced pressure. The product was purified by silica gel column chromatography, using 20% ether in petroleum ether (40–60 °C), to afford the title compound as a white solid in 81% yield (0.288 g). Single crystals were achieved by crystallization from its ethanol solution (El-Brollosy, 2007).

### Refinement

Carbon-bound H-atoms were placed in calculated positions [C—H = 0.95 to 0.99 Å, *U*_iso_(H) = 1.2*U*_eq_(C)] and were included in the refinement in the riding model approximation. The amino H-atom was refined freely.

### Crystal data

**Table:**

C_19_H_17_ClN_2_O_3_	*Z* = 2
*M**_r_* = 356.80	*F*(000) = 372
Triclinic, *P*1	*D*_x_ = 1.431 Mg m^−^^3^
Hall symbol: -P 1	Mo *K*α radiation, λ = 0.71073 Å
*a* = 7.6179 (3) Å	Cell parameters from 5016 reflections
*b* = 9.8168 (4) Å	θ = 2.4–27.5°
*c* = 11.7009 (6) Å	µ = 0.25 mm^−^^1^
α = 73.937 (4)°	*T* = 100 K
β = 83.651 (3)°	Prism, colourless
γ = 80.942 (3)°	0.35 × 0.30 × 0.15 mm
*V* = 828.31 (6) Å^3^	

### Data collection

**Table:**

Agilent SuperNova Dual diffractometer with an Atlas detector	3817 independent reflections
Radiation source: SuperNova (Mo) X-ray Source	3107 reflections with *I* > 2σ(*I*)
Mirror monochromator	*R*_int_ = 0.040
Detector resolution: 10.4041 pixels mm^-1^	θ_max_ = 27.6°, θ_min_ = 2.4°
ω scan	*h* = −9→9
Absorption correction: multi-scan (*CrysAlis PRO*; Agilent, 2011)	*k* = −12→12
*T*_min_ = 0.904, *T*_max_ = 1.000	*l* = −15→15
13263 measured reflections	

### Refinement

**Table:**

Refinement on *F*^2^	Primary atom site location: structure-invariant direct methods
Least-squares matrix: full	Secondary atom site location: difference Fourier map
*R*[*F*^2^ > 2σ(*F*^2^)] = 0.041	Hydrogen site location: inferred from neighbouring sites
*wR*(*F*^2^) = 0.114	H atoms treated by a mixture of independent and constrained refinement
*S* = 1.04	*w* = 1/[σ^2^(*F*_o_^2^) + (0.0527*P*)^2^ + 0.2968*P*] where *P* = (*F*_o_^2^ + 2*F*_c_^2^)/3
3817 reflections	(Δ/σ)_max_ = 0.001
231 parameters	Δρ_max_ = 0.35 e Å^−^^3^
0 restraints	Δρ_min_ = −0.27 e Å^−^^3^

### Special details

**Table:**

Geometry. All e.s.d.'s (except the e.s.d. in the dihedral angle between two l.s. planes) are estimated using the full covariance matrix. The cell e.s.d.'s are taken into account individually in the estimation of e.s.d.'s in distances, angles and torsion angles; correlations between e.s.d.'s in cell parameters are only used when they are defined by crystal symmetry. An approximate (isotropic) treatment of cell e.s.d.'s is used for estimating e.s.d.'s involving l.s. planes.
Refinement. Refinement of *F*^2^ against ALL reflections. The weighted *R*-factor *wR* and goodness of fit *S* are based on *F*^2^, conventional *R*-factors *R* are based on *F*, with *F* set to zero for negative *F*^2^. The threshold expression of *F*^2^ > σ(*F*^2^) is used only for calculating *R*-factors(gt) *etc*. and is not relevant to the choice of reflections for refinement. *R*-factors based on *F*^2^ are statistically about twice as large as those based on *F*, and *R*- factors based on ALL data will be even larger.

### Fractional atomic coordinates and isotropic or equivalent isotropic displacement parameters (Å^2^)

**Table:**

	*x*	*y*	*z*	*U*_iso_*/*U*_eq_	
Cl1	0.38928 (5)	0.76323 (5)	−0.23450 (4)	0.02434 (13)	
N1	0.49947 (18)	0.56212 (15)	0.32224 (13)	0.0176 (3)	
H1n	0.570 (3)	0.554 (2)	0.377 (2)	0.028 (5)*	
N2	0.21281 (17)	0.53780 (14)	0.28304 (12)	0.0155 (3)	
O1	0.69610 (15)	0.66966 (14)	0.17823 (11)	0.0257 (3)	
O2	0.30631 (15)	0.46261 (13)	0.47243 (10)	0.0204 (3)	
O3	−0.00414 (15)	0.38341 (12)	0.28957 (10)	0.0204 (3)	
C1	0.5525 (2)	0.62527 (18)	0.20503 (15)	0.0184 (3)	
C2	0.3372 (2)	0.51704 (17)	0.36544 (15)	0.0161 (3)	
C3	0.2530 (2)	0.59293 (16)	0.16058 (14)	0.0149 (3)	
C4	0.1268 (2)	0.60638 (18)	0.07786 (15)	0.0185 (3)	
H4	0.0116	0.5798	0.1047	0.022*	
C5	0.1707 (2)	0.65856 (18)	−0.04289 (15)	0.0190 (3)	
H5	0.0858	0.6671	−0.0990	0.023*	
C6	0.3390 (2)	0.69845 (17)	−0.08203 (15)	0.0182 (3)	
C7	0.4642 (2)	0.68767 (17)	−0.00267 (15)	0.0180 (3)	
H7	0.5784	0.7160	−0.0303	0.022*	
C8	0.4207 (2)	0.63424 (17)	0.11936 (15)	0.0159 (3)	
C9	0.0336 (2)	0.50004 (17)	0.32680 (15)	0.0173 (3)	
H9A	0.0235	0.4758	0.4150	0.021*	
H9B	−0.0559	0.5839	0.2974	0.021*	
C10	0.1126 (2)	0.25517 (18)	0.33274 (16)	0.0228 (4)	
H10A	0.2369	0.2763	0.3130	0.027*	
H10B	0.0950	0.1852	0.2896	0.027*	
C11	0.0895 (2)	0.18595 (17)	0.46551 (16)	0.0193 (4)	
C12	0.2574 (2)	0.0954 (2)	0.51400 (17)	0.0265 (4)	
H12A	0.2266	0.0100	0.5749	0.040*	
H12B	0.3217	0.1506	0.5495	0.040*	
H12C	0.3332	0.0666	0.4491	0.040*	
C13	−0.0679 (2)	0.20299 (18)	0.52568 (16)	0.0211 (4)	
H13	−0.1594	0.2663	0.4818	0.025*	
C14	−0.1176 (2)	0.13561 (18)	0.65213 (16)	0.0219 (4)	
C15	0.0034 (3)	0.0873 (2)	0.74056 (17)	0.0277 (4)	
H15	0.1236	0.1051	0.7217	0.033*	
C16	−0.0489 (3)	0.0139 (2)	0.85514 (18)	0.0345 (5)	
H16	0.0369	−0.0210	0.9131	0.041*	
C17	−0.2235 (3)	−0.0093 (2)	0.88651 (19)	0.0370 (5)	
H17	−0.2577	−0.0612	0.9651	0.044*	
C18	−0.3487 (3)	0.0438 (2)	0.8023 (2)	0.0346 (5)	
H18	−0.4702	0.0311	0.8235	0.042*	
C19	−0.2959 (2)	0.11572 (19)	0.68646 (18)	0.0267 (4)	
H19	−0.3827	0.1522	0.6294	0.032*	

### Atomic displacement parameters (Å^2^)

**Table:**

	*U*^11^	*U*^22^	*U*^33^	*U*^12^	*U*^13^	*U*^23^
Cl1	0.0269 (2)	0.0308 (2)	0.0125 (2)	−0.00363 (18)	−0.00140 (16)	−0.00102 (17)
N1	0.0145 (7)	0.0237 (7)	0.0145 (7)	−0.0037 (6)	−0.0058 (6)	−0.0025 (6)
N2	0.0141 (6)	0.0203 (7)	0.0117 (7)	−0.0025 (5)	−0.0029 (5)	−0.0025 (5)
O1	0.0153 (6)	0.0385 (7)	0.0207 (7)	−0.0082 (5)	−0.0041 (5)	0.0004 (6)
O2	0.0193 (6)	0.0284 (6)	0.0130 (6)	−0.0051 (5)	−0.0049 (5)	−0.0020 (5)
O3	0.0241 (6)	0.0221 (6)	0.0160 (6)	−0.0070 (5)	−0.0061 (5)	−0.0025 (5)
C1	0.0161 (8)	0.0212 (8)	0.0170 (9)	−0.0019 (6)	−0.0026 (6)	−0.0031 (7)
C2	0.0170 (7)	0.0171 (8)	0.0152 (8)	−0.0015 (6)	−0.0045 (6)	−0.0052 (6)
C3	0.0166 (7)	0.0147 (7)	0.0130 (8)	−0.0012 (6)	−0.0025 (6)	−0.0029 (6)
C4	0.0172 (8)	0.0222 (8)	0.0164 (9)	−0.0041 (6)	−0.0036 (6)	−0.0038 (7)
C5	0.0197 (8)	0.0215 (8)	0.0159 (9)	−0.0025 (7)	−0.0063 (6)	−0.0030 (7)
C6	0.0229 (8)	0.0189 (8)	0.0113 (8)	−0.0004 (7)	−0.0021 (6)	−0.0021 (6)
C7	0.0154 (7)	0.0201 (8)	0.0170 (9)	−0.0018 (6)	−0.0009 (6)	−0.0028 (7)
C8	0.0155 (7)	0.0169 (8)	0.0148 (8)	−0.0001 (6)	−0.0028 (6)	−0.0036 (6)
C9	0.0159 (7)	0.0218 (8)	0.0141 (8)	−0.0038 (6)	−0.0020 (6)	−0.0034 (7)
C10	0.0268 (9)	0.0221 (9)	0.0203 (9)	−0.0039 (7)	−0.0012 (7)	−0.0071 (7)
C11	0.0228 (8)	0.0168 (8)	0.0197 (9)	−0.0045 (6)	−0.0059 (7)	−0.0044 (7)
C12	0.0263 (9)	0.0284 (9)	0.0244 (10)	0.0015 (7)	−0.0051 (7)	−0.0081 (8)
C13	0.0228 (8)	0.0186 (8)	0.0209 (9)	−0.0024 (7)	−0.0069 (7)	−0.0019 (7)
C14	0.0279 (9)	0.0163 (8)	0.0217 (9)	−0.0037 (7)	0.0006 (7)	−0.0058 (7)
C15	0.0339 (10)	0.0279 (10)	0.0212 (10)	−0.0006 (8)	−0.0028 (8)	−0.0077 (8)
C16	0.0504 (12)	0.0326 (11)	0.0189 (10)	0.0022 (9)	−0.0042 (9)	−0.0078 (8)
C17	0.0606 (14)	0.0278 (10)	0.0218 (11)	−0.0097 (10)	0.0097 (10)	−0.0082 (8)
C18	0.0408 (11)	0.0285 (10)	0.0380 (12)	−0.0131 (9)	0.0149 (9)	−0.0167 (9)
C19	0.0298 (9)	0.0224 (9)	0.0297 (11)	−0.0045 (7)	0.0009 (8)	−0.0105 (8)

### Geometric parameters (Å, º)

**Table:**

Cl1—C6	1.7412 (17)	C9—H9B	0.9900
N1—C2	1.375 (2)	C10—C11	1.516 (2)
N1—C1	1.383 (2)	C10—H10A	0.9900
N1—H1n	0.85 (2)	C10—H10B	0.9900
N2—C2	1.379 (2)	C11—C13	1.333 (2)
N2—C3	1.402 (2)	C11—C12	1.507 (2)
N2—C9	1.471 (2)	C12—H12A	0.9800
O1—C1	1.2181 (19)	C12—H12B	0.9800
O2—C2	1.2298 (19)	C12—H12C	0.9800
O3—C9	1.4109 (19)	C13—C14	1.478 (2)
O3—C10	1.423 (2)	C13—H13	0.9500
C1—C8	1.472 (2)	C14—C15	1.395 (3)
C3—C8	1.395 (2)	C14—C19	1.401 (2)
C3—C4	1.405 (2)	C15—C16	1.383 (3)
C4—C5	1.385 (2)	C15—H15	0.9500
C4—H4	0.9500	C16—C17	1.378 (3)
C5—C6	1.391 (2)	C16—H16	0.9500
C5—H5	0.9500	C17—C18	1.386 (3)
C6—C7	1.376 (2)	C17—H17	0.9500
C7—C8	1.400 (2)	C18—C19	1.391 (3)
C7—H7	0.9500	C18—H18	0.9500
C9—H9A	0.9900	C19—H19	0.9500
			
C2—N1—C1	127.24 (14)	O3—C10—C11	115.55 (14)
C2—N1—H1n	113.6 (14)	O3—C10—H10A	108.4
C1—N1—H1n	119.0 (14)	C11—C10—H10A	108.4
C2—N2—C3	121.94 (13)	O3—C10—H10B	108.4
C2—N2—C9	118.11 (13)	C11—C10—H10B	108.4
C3—N2—C9	119.94 (13)	H10A—C10—H10B	107.5
C9—O3—C10	113.33 (12)	C13—C11—C12	126.82 (17)
O1—C1—N1	121.37 (15)	C13—C11—C10	120.77 (15)
O1—C1—C8	124.46 (16)	C12—C11—C10	112.35 (15)
N1—C1—C8	114.16 (14)	C11—C12—H12A	109.5
O2—C2—N1	120.90 (14)	C11—C12—H12B	109.5
O2—C2—N2	122.60 (14)	H12A—C12—H12B	109.5
N1—C2—N2	116.49 (14)	C11—C12—H12C	109.5
C8—C3—N2	120.01 (14)	H12A—C12—H12C	109.5
C8—C3—C4	119.13 (15)	H12B—C12—H12C	109.5
N2—C3—C4	120.86 (14)	C11—C13—C14	128.07 (16)
C5—C4—C3	119.85 (15)	C11—C13—H13	116.0
C5—C4—H4	120.1	C14—C13—H13	116.0
C3—C4—H4	120.1	C15—C14—C19	117.21 (17)
C4—C5—C6	120.04 (15)	C15—C14—C13	123.94 (16)
C4—C5—H5	120.0	C19—C14—C13	118.85 (16)
C6—C5—H5	120.0	C16—C15—C14	121.07 (19)
C7—C6—C5	121.20 (16)	C16—C15—H15	119.5
C7—C6—Cl1	120.11 (13)	C14—C15—H15	119.5
C5—C6—Cl1	118.70 (13)	C17—C16—C15	121.0 (2)
C6—C7—C8	118.90 (15)	C17—C16—H16	119.5
C6—C7—H7	120.5	C15—C16—H16	119.5
C8—C7—H7	120.5	C16—C17—C18	119.32 (19)
C3—C8—C7	120.88 (14)	C16—C17—H17	120.3
C3—C8—C1	119.84 (15)	C18—C17—H17	120.3
C7—C8—C1	119.26 (14)	C17—C18—C19	119.79 (19)
O3—C9—N2	112.34 (13)	C17—C18—H18	120.1
O3—C9—H9A	109.1	C19—C18—H18	120.1
N2—C9—H9A	109.1	C18—C19—C14	121.51 (19)
O3—C9—H9B	109.1	C18—C19—H19	119.2
N2—C9—H9B	109.1	C14—C19—H19	119.2
H9A—C9—H9B	107.9		
			
C2—N1—C1—O1	175.02 (16)	C6—C7—C8—C1	−178.68 (15)
C2—N1—C1—C8	−4.4 (2)	O1—C1—C8—C3	−173.69 (16)
C1—N1—C2—O2	−179.73 (15)	N1—C1—C8—C3	5.7 (2)
C1—N1—C2—N2	−0.5 (2)	O1—C1—C8—C7	4.7 (3)
C3—N2—C2—O2	−176.52 (14)	N1—C1—C8—C7	−175.91 (14)
C9—N2—C2—O2	3.4 (2)	C10—O3—C9—N2	61.77 (17)
C3—N2—C2—N1	4.2 (2)	C2—N2—C9—O3	−113.36 (15)
C9—N2—C2—N1	−175.90 (13)	C3—N2—C9—O3	66.52 (18)
C2—N2—C3—C8	−2.7 (2)	C9—O3—C10—C11	70.12 (18)
C9—N2—C3—C8	177.40 (14)	O3—C10—C11—C13	28.0 (2)
C2—N2—C3—C4	176.78 (15)	O3—C10—C11—C12	−154.74 (14)
C9—N2—C3—C4	−3.1 (2)	C12—C11—C13—C14	−1.7 (3)
C8—C3—C4—C5	0.8 (2)	C10—C11—C13—C14	175.12 (16)
N2—C3—C4—C5	−178.76 (15)	C11—C13—C14—C15	25.9 (3)
C3—C4—C5—C6	−0.4 (3)	C11—C13—C14—C19	−152.81 (18)
C4—C5—C6—C7	−0.2 (3)	C19—C14—C15—C16	4.5 (3)
C4—C5—C6—Cl1	179.88 (13)	C13—C14—C15—C16	−174.27 (17)
C5—C6—C7—C8	0.6 (3)	C14—C15—C16—C17	−2.3 (3)
Cl1—C6—C7—C8	−179.53 (12)	C15—C16—C17—C18	−1.0 (3)
N2—C3—C8—C7	179.11 (14)	C16—C17—C18—C19	2.0 (3)
C4—C3—C8—C7	−0.4 (2)	C17—C18—C19—C14	0.3 (3)
N2—C3—C8—C1	−2.5 (2)	C15—C14—C19—C18	−3.5 (3)
C4—C3—C8—C1	178.01 (15)	C13—C14—C19—C18	175.33 (16)
C6—C7—C8—C3	−0.3 (2)		

### Hydrogen-bond geometry (Å, º)

Cg1 is the centroid of the C14–C19 benzene ring.

**Table:**

*D*—H···*A*	*D*—H	H···*A*	*D*···*A*	*D*—H···*A*
N1—H1*n*···O2^i^	0.85 (2)	2.05 (2)	2.8932 (18)	168.9 (19)
C4—H4···O1^ii^	0.95	2.57	3.382 (2)	144
C5—H5···O3^iii^	0.95	2.57	3.427 (2)	150
C9—H9*B*···O1^ii^	0.99	2.38	3.232 (2)	144
C10—H10*A*···*Cg*1^iv^	0.99	2.69	3.612 (2)	154

Symmetry codes: (i) −*x*+1, −*y*+1, −*z*+1; (ii) *x*−1, *y*, *z*; (iii) −*x*, −*y*+1, −*z*; (iv) −*x*, −*y*, −*z*+1.
